# Decreasing Inappropriate Telemetry Use via Nursing-Driven Checklist and Electronic Health Record Order Set

**DOI:** 10.7759/cureus.28999

**Published:** 2022-09-10

**Authors:** Michelle Knees, Katarzyna Mastalerz, Joseph Simonetti, Andrew Berry

**Affiliations:** 1 Division of Hospital Medicine, University of Colorado Anschutz Medical Campus, Aurora, USA; 2 Denver-Seattle Center of Innovation for Veteran-Centered and Value-Driven Care, Rocky Mountain Regional Veterans Affairs Medical Center, Aurora, USA; 3 Hospital Medicine Group, Rocky Mountain Regional Veterans Affairs Medical Center, Aurora, USA

**Keywords:** telemetry overuse, patient safety, quality improvement, plan do study act, pdsa cycle, nursing checklist, electronic health record (ehr)

## Abstract

Introduction: Telemetry is ubiquitous in many hospitals despite widely acknowledged limitations, waste, and potential harm associated with inappropriate use. To curb overuse, guidelines such as the 2017 American Heart Association/American College of Cardiology (AHA/ACC) continuous telemetry monitoring practice standards have outlined appropriate telemetry use standards. This study aimed to perform two “plan-do-study-act” (PDSA) cycles and assess whether a nursing (RN)-driven checklist addressing appropriate telemetry use, combined with just-in-time education delivered via an electronic health record (EHR) order set modification, was efficacious in reducing inappropriate telemetry use within a level 1a Veterans Health Administration hospital.

Methods: This is a quality improvement intervention study that took place between March 2019 and August 2020. Three cohorts were sequentially studied: a control cohort without any intervention (n = 100), a cohort with only the RN-driven checklist (n = 100), and a cohort with both the RN-driven checklist and an EHR order set modification that provided just-in-time education about telemetry indications (n = 100). Telemetry records were reviewed by a physician to determine indication, duration for each telemetry order, and appropriateness. An order was deemed “appropriate” if it met AHA/ACC classification grade I (telemetry recommended) or IIa/b (telemetry may be considered) and “inappropriate” if it fell under class III (telemetry not recommended). Data were compared between the control cohort and the two intervention cohorts, as well as between intervention cohorts, using Pearson chi-square analysis. A p-value < 0.05 was considered statistically significant.

Results: Within the control group, 37% of telemetry orders were deemed inappropriate. After implementation of the RN checklist, a non-statistically significant lower proportion (26%) of orders was deemed inappropriate (p = 0.09). Implementation of the RN checklist, along with the EHR order set, was associated with a significantly lower proportion of inappropriate orders (17%) in comparison to the control cohort (p = 0.001) but not in comparison to the RN checklist cohort (p = 0.12). There was no significant difference in the duration of telemetry use across cohorts.

Conclusions: An RN-driven checklist and EHR telemetry order set modification were associated with a decrease in inappropriate telemetry use from 37% to 17%. By prompting the review of telemetry orders via a daily nursing checklist reviewed during bedside interdisciplinary rounds, clinicians received reinforcement regarding appropriate telemetry indications. This education was strengthened by the just-in-time training provided via the EHR order set.

## Introduction

Telemetry was introduced to hospitals in the 1960s to monitor cardiac intensive care unit patients [1]. Since then, it has become widely used in many hospitals to monitor a variety of patient conditions. However, appropriate usage varies significantly. A 2018 study at a tertiary care hospital found only 24% of telemetry days were appropriate and a separate study found up to 43% of monitored patients lacked an appropriate indication [2,3]. Although reported telemetry costs differ significantly between institutions, reported daily expenses range from $34 to $1400 per patient per 24 hours [2,4]. Importantly, these estimates do not account for additional costs incurred from artifact data and incidental findings, which can lead to unnecessary evaluations and procedures for patients. Additionally, inappropriate telemetry use contributes to alarm fatigue. A 12-day alarm system analysis conducted at a large academic hospital in 2014 found that non-intensive care beds had, on average, 350 alerts per day [5]. Between 2005 and 2008, 566 deaths were attributed to alarm fatigue. Accordingly, the Emergency Care Research Institute has listed alarm fatigue within its top 10 health technology hazards every year since its creation in 2007 [6].

There are multiple reasons for telemetry overuse. In addition to variable physician knowledge about appropriate telemetry indications, medical teams are frequently unaware their patients are being monitored. A 2016 study by Sharma et al. found that 47% of patients reviewed were monitored on telemetry. Of these patients, providers were only aware they were being monitored 74% of the time and only 58% of providers were able to provide guideline-appropriate indications for monitoring [7]. In an attempt to curb inappropriate utilization, the American Heart Association (AHA) and American College of Cardiology (ACC) updated their recommendations for continuous telemetry monitoring in 2017 [8]. Since then, several prominent medical journals and societies, including the Journal of the American Medical Association (JAMA) and the Society of Hospital Medicine (SHM), have published blueprints and recommendations for decreasing inappropriate telemetry monitoring [3,9].

Our study utilized a nursing (RN)-driven checklist and an electronic health record (EHR) order set modification to perform two plan-do-study-act (PDSA) cycles to decrease inappropriate telemetry utilization within a level 1a Veterans Health Administration hospital.

This article was previously presented as a poster at the Society of Hospital Medicine Converge 2022 annual meeting on April 8, 2022.

## Materials and methods

Study design and population

We conducted a quality improvement intervention study that relied on a retrospective chart review. This study was conducted at the Rocky Mountain Regional VA Medical Center, a level 1A teaching hospital. It was submitted for Institutional Review Board review and was deemed exempt under quality improvement guidelines. Inclusion criteria for chart review included patient age ≥ 18 years, alive during admission where telemetry was utilized, admission to the general medical floor, and admission to an internal medicine service. Exclusion criteria included coronavirus disease 2019 (COVID-19) diagnosis, age < 18 years, pregnancy at the time of admission, and death during admission. Charts were then analyzed sequentially until 100 patients were reviewed in each of the three cohorts. This first cohort analysis period was from March 1, 2019, through March 28, 2019, the second cohort analysis period was from July 1, 2019, through July 31, 2019, and the third cohort analysis period was from July 1, 2020, through August 18, 2020.

Data

Three cohorts were sequentially studied. For each cohort studied, telemetry records were obtained from the central telemetry office. Each patient chart was reviewed by an internal medicine physician (Michelle Knees, author), to determine the indication for the initial telemetry order. Using the 2017 AHA/ACC continuous telemetry appropriate use guidelines, an order was deemed “appropriate” if it met AHA class I guidelines (should be performed) or class IIa/b guidelines (reasonable to perform or may be considered). If the telemetry order indication fell under class III guidelines (monitoring has no benefit or may be harmful), it was marked as “inappropriate.” If the telemetry indication was not addressed by AHA/ACC guidelines, the reviewing physician analyzed the chart to determine the appropriateness of the order based on patient stability as recommended by the AHA/ACC [8].

Next, telemetry duration was recorded in the “number of midnights” based on the date of order initiation and discontinuation. The patient’s chart was then reviewed to ensure that telemetry monitoring was required throughout the course of an active telemetry order, again based on stability and AHA/ACC guidelines.

Intervention details

**Figure 1:**
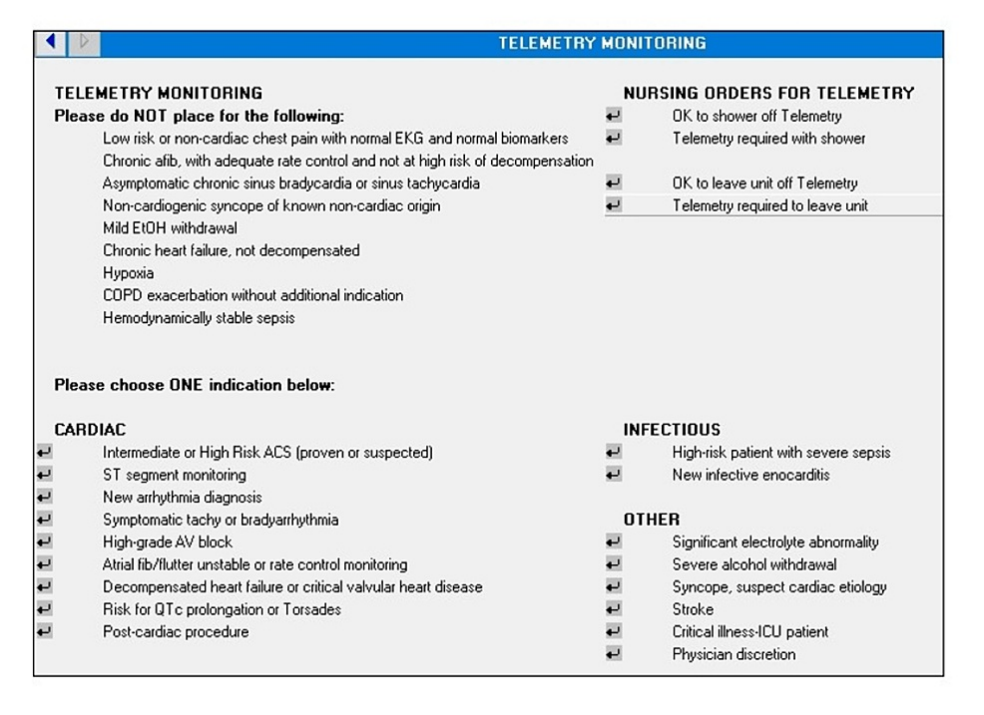
Electronic health record telemetry order set The order set was designed to provide just-in-time education by prompting physicians with indications for appropriate telemetry use. COPD: chronic obstructive pulmonary disease; ACS: acute coronary syndrome.

Three cohorts were identified and patients were analyzed consecutively until an "n" of 100 was reached within each cohort. The control cohort did not have any intervention (n = 100; March 1, 2019-March 28, 2019). The second cohort reviewed (PDSA cycle 1) had only an RN-driven checklist (n = 100; July 1, 2019-July 31, 2019), which, among other items, prompted nurses to address active telemetry orders during bedside interdisciplinary rounds as part of their standardized report. Nursing education started on May 15, 2019, and checklist implementation occurred on June 26, 2019. The third cohort reviewed (PDSA cycle 2) had both the RN-driven checklist and an EHR order set modification, as seen in Figure 1, which provided just-in-time education about telemetry indications (n = 100; July 1, 2020-August 18, 2020) based on the AHA/ACC 2017 guidelines [8]. It was also designed to auto-expire after 72 hours to prompt intentional telemetry continuation in patients who need longer monitoring. PDSA cycle 2 occurred one year after PDSA cycle 1 as it was a resident-led quality improvement project and the resident was not at the Veterans Affairs (VA) again until the following academic year. The checklist was maintained during this time.

Statistical methods

To have 80% power and appreciate a 30% reduction in initial telemetry orders, 72 patients were needed for each cohort. Therefore, 100 patients were chosen for each cohort to ensure that the study would be appropriately powered. We compared the proportion of patients in each cohort who were deemed to have an inappropriate medical indication for telemetry monitoring via Pearson chi-square analysis. We limited our analysis of telemetry duration only to charts that were initially deemed appropriate.

## Results

**Figure 2:**
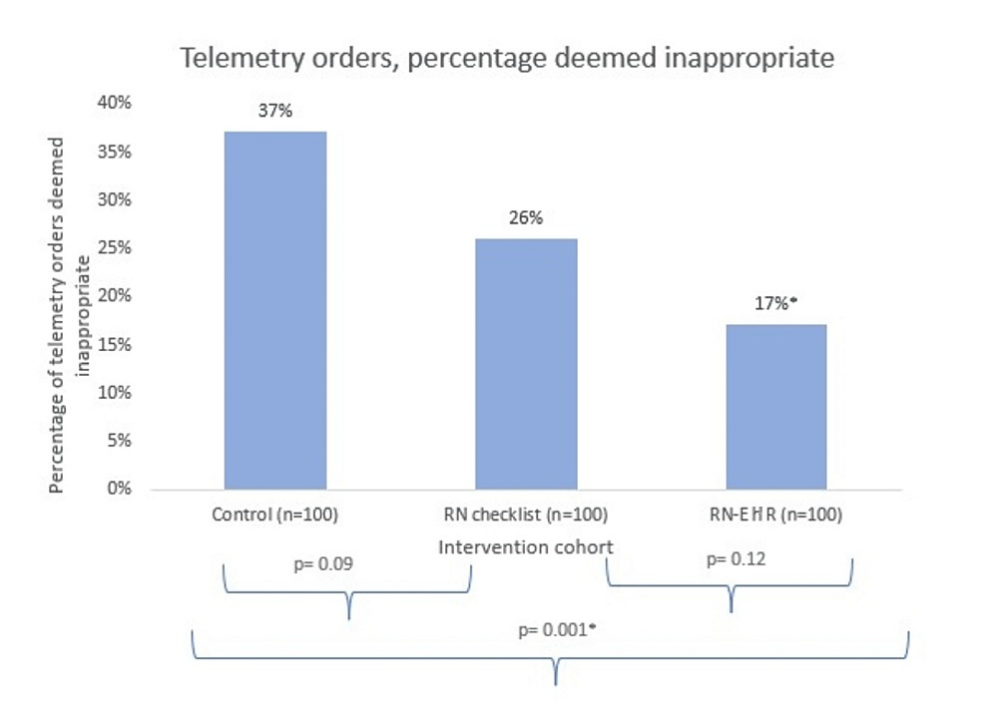
Percentage of telemetry orders that were deemed "inappropriate" within the control, RN checklist, and RN-EHR cohorts There was a statistically significant difference in the number of inappropriate telemetry orders between the control cohort and the combined RN-EHR cohort (p = 0.001). RN: nursing; EHR: electronic health record.

Within the control group, 37% of telemetry orders were deemed inappropriate. After implementation of the RN checklist, a non-statistically significant lower proportion (26%) of orders was deemed inappropriate (p = 0.09). As seen in Figure 2, implementation of the RN checklist, along with the EHR order set, was associated with a significantly lower proportion of inappropriate orders (17%) in comparison to the control cohort (p = 0.001), but not in comparison to the RN checklist cohort (p = 0.12).

**Figure 3:**
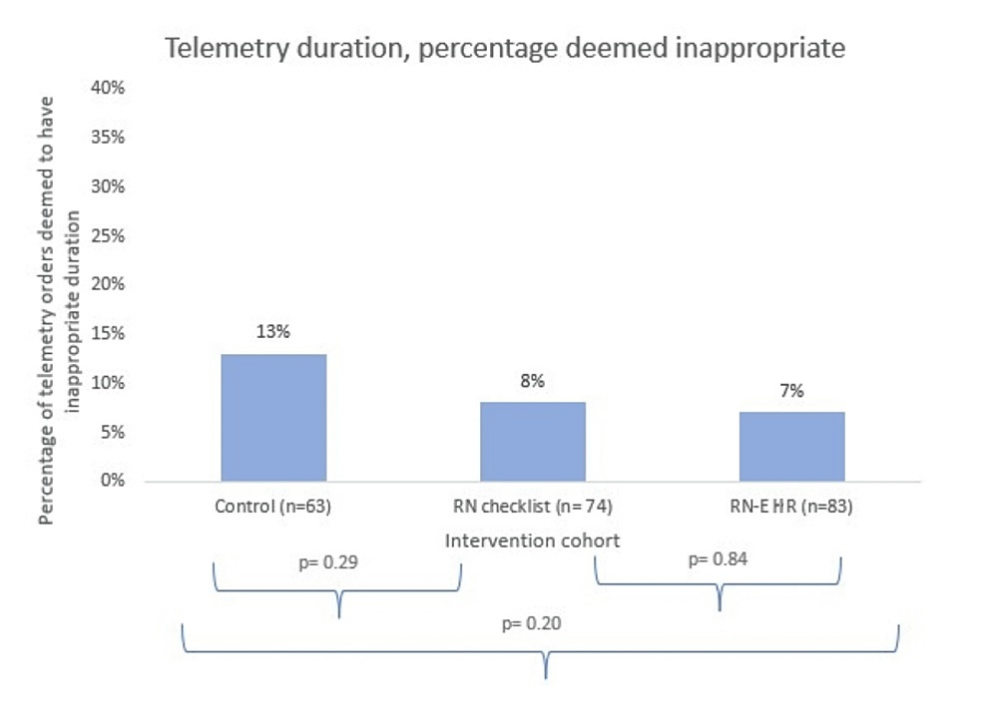
Percentage of telemetry orders that were found to be inappropriate in duration within the control, RN checklist, and RN-EHR cohorts There were no statistically significant differences in inappropriate telemetry duration between the cohorts. RN: nursing; EHR: electronic health record.

The same comparison was made between the control and intervention cohorts for inappropriate telemetry duration; only appropriate initial telemetry orders were analyzed for the inappropriate duration. As seen in Figure 3, within the control group, 13% of appropriate telemetry orders were deemed to have an inappropriate duration (mean duration: 2.16 days; median/mode duration: one day). Compared to this, no statistically significant difference was found within the RN checklist cohort where 8% of appropriate telemetry orders were deemed to have an inappropriate duration (mean duration: 2.06 days; median and mode duration: one day; p = 0.29), or within the RN/EHR checklist cohort where 7% of appropriate telemetry orders were deemed to have an inappropriate duration (mean duration: 2.01 days; median and mode duration: one day; p = 0.20). Comparison between the two intervention groups (RN and RN/EHR) also did not meet statistical significance (p = 0.84).

## Discussion

By utilizing two PDSA cycles, our institution initiated a nursing-driven checklist and EHR telemetry order set modification, which was associated with a statistically and clinically significant 54% reduction in inappropriate telemetry ordering. There are several potential mechanisms by which these interventions could have reduced inappropriate telemetry use. A prior study found that hospitalist-led discussion about telemetry use during daily rounds led to a reduction in length of stay and cost for telemetry bed utilization [10]. While we did not see a reduction in overall telemetry duration and did not analyze cost savings, we did see a non-statistically significant 30% decrease in telemetry misuse when we asked nurses to discuss telemetry orders during daily multidisciplinary rounds. We hypothesize that daily discussion of telemetry provided physician education regarding appropriate telemetry use and impacted their future ordering practices. The just-in-time education provided by the EHR order set may have further provided real-time clinician education regarding appropriate telemetry usage. Other studies have found that EHR order set configurations can be used to discourage ordering by hiding automated orders, restricting ordering privileges to certain providers, or creating alerts that ask providers to verify orders [11]. Less research has been done regarding just-in-time education via EHR order sets, but we hypothesize that by providing common indications for telemetry ordering, as well as by highlighting common inappropriate ordering patterns, we were able to prompt physicians into decreasing telemetry ordering for inappropriate clinical indications.

While there was not a statistically significant decrease in telemetry duration, we have several proposed explanations. Within the control group, only 13% of appropriate orders were utilized for an inappropriate duration. Additionally, the mean duration was only 2.16 days, and the median and mode durations were only one day. A large limitation of our data is that telemetry duration can only be counted in midnights, with one midnight equaling one day. Our telemetry office records the day that someone is placed on telemetry and the day it is removed; thus, telemetry placed at 00:01 on day one and removed at 23:59 on day two would only count as one day, even though this more closely represents two clinical days. Additionally, in length-of-use analyses, power is limited due to smaller sample size, and to see a difference when the duration of inappropriate use was only 13% and the mean duration was only two days would likely require a much larger sample size. Had the initial telemetry duration exceeded 72 hours, the 72-hour auto-expiration in the EHR order set may have become more clinically and statistically relevant. Similarly, the RN checklist is utilized only during morning rounds, which generally occur one midnight after telemetry has been ordered; given that the control data had a mean duration of 2.16 days, decreasing this further via the nursing intervention would be challenging.

Our analysis has several additional limitations. The time periods of analysis differ between cohorts; the first analysis occurred in March 2019, the second in July 2019, and the third in July 2020. Because the time periods differed, there may be residual confounding, which we were unable to account for in our analyses. A larger limitation is the impact of the COVID-19 pandemic. The first two study cycles occurred in March and July 2019 while the third cycle occurred in July 2020. Other studies have found that inappropriate telemetry use increased during the early phase of the pandemic; thus, we attempted to limit the impact of COVID-19 on our analysis by excluding patients with COVID-19 infection from our analyses [12]. A final limitation is that we only reviewed charts of patients who had an order for telemetry. It is possible that while incorrect telemetry orders did decrease across the time periods analyzed, patients who should have received telemetry monitoring did not have it ordered.

Additional avenues of study should include the following: an effectiveness study with a control group to ensure that the intervention is effective; replicability of our intervention at other institutions; analysis of rapid responses, code blues, patient transfers to intensive care units, and patient deaths to ensure no unintended negative patient safety effects; cost analysis to quantify the amount of money saved by decreasing inappropriate telemetry use; the impact of decreased telemetry use on patient length of stay; and impact of decreased telemetry use on patient and nursing satisfaction.

## Conclusions

Telemetry is a powerful tool for monitoring patients at high risk of cardiac decompensation. However, it is also associated with excess costs, alarm fatigue, and false positive findings and should only be utilized in patients who will benefit from continuous cardiac monitoring; decreasing inappropriate telemetry use is, therefore, important from a financial and patient safety standpoint. Several major medical journals and societies, including JAMA, SHM, AHA, and ACC, have published guidelines to help hospitals devise interventions to curtail inappropriate monitoring.

This quality improvement intervention utilized the AHA and ACC telemetry guidelines to perform two PDSA cycles, including a nursing-driven checklist and EHR order set modification. We were able to decrease our telemetry misuse from 37% to 17%, which is both statistically and clinically significant. We hypothesize this decrease was largely driven by just-in-time education provided by the EHR order set, which was solidified during discussions about appropriate telemetry use during morning multidisciplinary rounds. Given the relative affordability, other institutions with high rates of telemetry misuse could consider similar interventions.
